# Neuronal guidance behaviours: the primary cilium perspective

**DOI:** 10.3389/fcell.2025.1612555

**Published:** 2025-06-30

**Authors:** Melody Atkins, Coralie Fassier, Xavier Nicol

**Affiliations:** Sorbonne Université, Institut National de la Santé et de la Recherche Médicale (INSERM), Centre National de la Recherche Scientifique (CNRS), Institut de la Vision, Paris, France

**Keywords:** neuronal guidance, primary cilium, neuronal migration, axon guidance, signalling pathways

## Abstract

The establishment of functional neuronal circuits critically relies on the ability of developing neurons to accurately sense and integrate a variety of guidance signals from their surrounding environment. Such signals are indeed crucial during key steps of neuronal circuit wiring, including neuronal migration and axon guidance, to guide developing neurons or extending axons towards their target destination in the developing brain. The growth cone, located at the tip of developing neurons, is a key subcellular structure in this process, that concentrates many different guidance receptors and signalling molecules and specialises in the probing and integration of extracellular signals into various guidance behaviours. Interestingly, the small primary cilium, long considered as a vestigial organelle, has progressively emerged as a cellular antenna specialised in cell signalling, and has been reported, just like the growth cone, to harbour a variety of guidance receptors. How primary cilium-elicited signals are then transduced into specific cellular processes to guide developing neurons and axons remains however obscure. In this review, we will summarise our emerging understanding of the role of primary cilium-elicited signalling pathways on neuronal guidance processes, by focusing on neuronal migration and axon guidance. We will highlight the primary cilium molecular diversity, and how it shapes the primary cilium functional versatility, allowing the ciliary compartment to instruct various guidance behaviours through the regulation of different cellular processes. We will moreover discuss current and future avenues of research, to unravel the different molecular effectors activated downstream of specific ciliary signals, and clues to be gained from studies performed in non-neuronal cells. Rising challenges of the field will also be addressed, such as the technical challenge induced by the dual subcellular localisation (*i.e*., ciliary and extra-ciliary) of many ciliary guidance receptors, and the importance of the development of new genetic/chemo-genetic/optogenetic tools. Finally, we will highlight the insight such studies will bring for our understanding of the aetiology of different disorders, including ciliopathies, neurodevelopmental and neurodegenerative disorders, but also cancer cell migration/invasion, which are associated with defective primary cilium formation and function.

## 1 Introduction

Neuronal guidance signalling encompasses all the signalling processes that ensure precise neuronal positioning and wiring (Yuasa-Kawada et al., 2022). Neuronal migration and axon pathfinding are two major steps of this guidance process. Newly generated neurons indeed migrate from their birthplace to their final destination in the developing brain and extend their growing axons towards the right synaptic targets. The neuron’s environment is a key ally in this developmental journey, as it provides different spatiotemporally-controlled guidance signals that enable developing neurons to ultimately integrate functional neuronal circuits. Depending on the neuronal subtype and/or the developmental stage, migration and axon navigation can occur either sequentially or concomitantly. Adding to this complexity, a same guidance signal can steer different populations of neurons and/or elicit different types of guidance behaviours (*e.g.*, neuronal migration or axon guidance), highlighting the importance for developing neurons to accurately sense and integrate multiple extracellular signals in order for accurate neural circuit wiring to occur.

Extracellular guidance cues are sensed by receptors/channels expressed at the surface of developing neurons and come in many different flavours. They can be chemical, including diffusible extracellular or cell-bound ligands (proteins, lipids, small molecules …), but also mechanical, or even electrical (Gangatharan et al., 2018; Medvedeva and Pierani, 2020; Dorskind and Kolodkin, 2021). The growth cone, that is formed at the tip of extending axons and migrating neurons alike, is known to express many guidance receptors and is extensively studied as a key structure specialised in the probing and integration of the extracellular environment (Lowery and Van Vactor, 2009; Stoeckli, 2018; Nakajima et al., 2024). Interestingly, developing neurons–as almost all vertebrate cells–possess another key subcellular compartment, the primary cilium (PC), that has progressively emerged as a cell antenna specialised in collecting signals from the environment. Indeed, mutations affecting the PC structure and/or function have been found to induce a group of developmental disorders termed ciliopathies. While the clinical manifestations of ciliopathies are multisystemic, and include retinopathy, obesity, diabetes, skeletal malformations, and hepatic disease, ciliopathies are also characterised by a wide range of neurodevelopmental defects, such as in the Joubert (JBTS), Meckel-Grüber (also called Meckel syndrome, MKS) or Bardet–Biedl syndromes (Reiter and Leroux, 2017; Andreu-Cervera et al., 2021; Karalis et al., 2022). These defects include brain malformations, ataxia, epilepsy, mental disability and highlight the importance of primary cilia in neuronal circuit wiring and function. Accordingly, recent studies have located several receptors/effectors of major guidance signalling pathways to the ciliary compartment (Higginbotham et al., 2012; Loukil et al., 2023). However, the precise signalling events elicited in response to guidance signals within the PC and transduced to downstream intracellular effectors in order to regulate neuronal guidance behaviours remain poorly understood.

In this review, we will summarise our current understanding of the role of PC-elicited signalling pathways on neuronal guidance processes, focusing on neuronal migration and axon guidance. We will highlight the importance of the molecular diversity of the ciliary compartment, and how it determines the functional versatility of PC signalling during neuronal guidance, regulating: **(i)** different guidance processes (*i.e.*, neuronal migration and axon navigation) sequentially or concomitantly, and **(ii)** different molecular mechanisms converging on a same guidance process (*e.g.*, neuronal migration). It is indeed important to bear in mind that the generic PC does not exist, and that ciliary composition is highly versatile, at different levels. First, **(i)** the PC protein composition varies throughout the lifespan of the cell: for example, the expression of the ciliary marker, adenylate cyclase 3 (AC3; *i.e.*, enzyme responsible for the cAMP cyclic nucleotide synthesis) is low in the embryonic brain, but increases during the first postnatal weeks, before decreasing again at later stages (Arellano et al., 2012). Ciliary protein composition is moreover **(ii)** highly dependent on the cell type, and depending on the cell type, **(iii)** a same ciliary protein can show different sub-ciliary localisation patterns (Hansen et al., 2022). We will moreover discuss current and future research avenues to unravel the many ramifications of molecular effectors activated downstream of specific PC-elicited guidance signals, and clues to be gained from studies performed in non-neuronal cells. Finally, we will highlight the insight such studies will bring for our understanding of ciliopathies, but also neurodevelopmental and neurodegenerative disorders or cancer cell migration, associated with defective PC formation and function.

## 2 The neuronal primary cilium: a signalling hub sensing environmental guidance cues

### 2.1 The primary cilium subcellular compartment

Primary cilia are small, microtubule-based structures that are contiguous with the plasma membrane and bud from the surface of almost all vertebrate cells. Observed as early as 1898 (Zimmermann, 1898), technical limitations have long relegated the PC to a vestigial organelle, until the development of transmission electron microscopy and the association made between primary cilia and ciliopathies gradually boosted our interest for this tiny organelle. Since then, ciliopathies have been reported one after the other, with the discovery of more and more ciliopathy-associated genes (Reiter and Leroux, 2017), the study of which has contributed to considerably increase our knowledge of the PC structure and function.

#### 2.1.1 The primary cilium structure and composition

**FIGURE 1:**
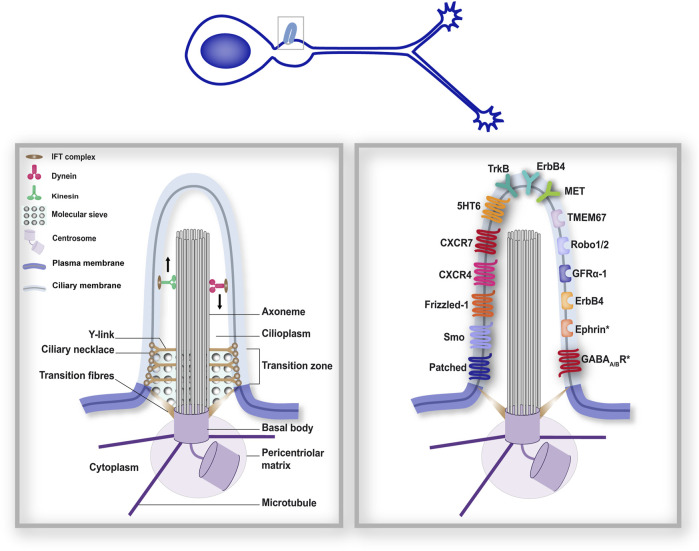
The primary cilium forms a distinct subcellular compartment that functions as a signalling hub. The structural organisation of the PC (left-hand boxed region) comprises different gating mechanisms that ensure a distinct protein composition of the ciliary compartment, in addition to its distinct lipidic composition. As a consequence, many membrane receptors have been reported at the surface of the PC. The right-hand boxed region depicts neuronal guidance-related membrane receptors reported at the surface of neuronal primary cilia during development (Rodriguez Gil and Greer, 2008; Williams et al., 2010; Petralia et al., 2011; Higginbotham et al., 2012; Toro-Tapia and Das, 2020). Receptors marked with an (*) were found in neuronal cilia postnatally (Loukil et al., 2023). Left- and right-hand boxed regions correspond to a higher magnification of the PC of the developing neuron depicted above.

The architecture of the PC has been extensively studied. The PC is organised by a modified mother centriole, called **the basal body**, from which the ciliary microtubule core, called **the axoneme** (comprising nine microtubule doublets), extends, surrounded by the ciliary membrane (Figure 1). In mammalian neurons, the PC extends 2 to 12 μm from the cell surface, with a diameter ∼ 200–500 nm (DeMars et al., 2023; Macarelli et al., 2023)**.** Two main ciliogenesis pathways have been described: the extracellular pathway, and the intracellular one, that is the most studied (Wang and Dynlacht, 2018; Hoffman and Prekeris, 2022; Zhao et al., 2023). While extracellular ciliogenesis occurs in most polarised epithelial cells, the intracellular pathway appears to be favoured by most other cell types (Sorokin, 1962; 1968; Molla-Herman et al., 2010; Labat-de-Hoz et al., 2021). In the intracellular pathway, ciliogenesis starts in the cytoplasm with the docking of the basal body to a large ciliary vesicle. The axoneme assembles from the basal body beneath this vesicle. As the axoneme extends, the ciliary vesicle expands to encapsulate the axoneme in a double membrane layer, with the ciliary membrane facing the axoneme and the ciliary sheat facing the cytoplasm. PC budding at the cell surface is then enabled by fusion of the ciliary sheat with the plasma membrane. Conversely, extracellular ciliogenesis is initiated by the docking of the basal body to the plasma membrane. As the axoneme extends from the basal body, the ciliary membrane is gradually formed from the plasma membrane. Whether in the extracellular or intracellular pathway, extension of the PC, in which translation does not occur, relies on a ciliary transport system, **the intraflagellar transport** (IFT), that uses the axoneme scaffold to provide all the building material required for membrane and axoneme extension, as well as for protein delivery and exit to and from the PC. IFT (Taschner and Lorentzen, 2016) is powered by the kinesin-II and dynein microtubule-based molecular motors for anterograde and retrograde transport along the axoneme, respectively. Trains of IFT particles, each composed of IFTA and IFTB subcomplexes, are assembled at the ciliary base and couple the molecular motors to the cargoes for ciliary trafficking to and from the PC tip.

#### 2.1.2 The primary cilium: a signalling hub

This IFT system is important not only for ciliogenesis, but also for PC function. Indeed, the wide range of ciliopathy-associated phenotypes and target organs–ranging from skeletal, heart, kidney, renal or retinal malfunction to brain malformations and cognitive defects–highlights the crucial involvement of the highly conserved PC in the regulation of cell signalling and function. The PC is indeed now well established as a signalling hub at the crossroads between various signalling pathways (Christensen et al., 2012; Hilgendorf et al., 2016; Pala et al., 2017; Wheway et al., 2018; Anvarian et al., 2019; Nishimura et al., 2019; Mill et al., 2023). The IFT transport machinery plays an important part in the concentration and trafficking into and out of the tiny ciliary volume of many membrane receptors (*e.g.*, G-protein coupled receptors, ion channels, extracellular matrix receptors, purinergic receptors …) and signalling molecules (*e.g.*, second messengers, soluble proteins …). Of note, the precise molecular mechanisms involved in these various trafficking events remain to be clarified, and an IFT-independent lateral diffusion of certain ciliary membrane receptors along the axoneme has also been proposed (Milenkovic et al., 2009; Ye et al., 2013). Proteomic studies performed in non-neuronal systems have nevertheless contributed to confirm the diversity of proteins concentrated within the ciliary volume and hint at the wide variety of processes in which the PC signalling hub is involved (Ishikawa et al., 2012; Mick et al., 2015; Hansen et al., 2024; Liu et al., 2024).

This dense and diverse protein composition is a key feature of the PC compartment, along with its lipidic composition, that is distinct from that of the plasma membrane (Nakatsu, 2015; Conduit and Vanhaesebroeck, 2020). Different gating mechanisms, based on evolutionarily-conserved domains located at the base of the PC, act in concert with the IFT to strictly restrict the exchanges between the cytoplasm and the cilioplasm (Jensen and Leroux, 2017; Park and Leroux, 2022; Moran et al., 2024).

At the very base of the PC, the **distal appendages (or transition fibres**, see Figure 1 **)** of the cell body connect the basal body to the ciliary membrane. IFT particles dock onto transition fibres before cargo trafficking to the ciliary compartment (Deane et al., 2001; Wei et al., 2013). Distal to the transition fibres, the **transition zone** is composed of Y-links that connect the axoneme to the ciliary membrane, and the ciliary necklace, comprising rows of membrane particles that encircle the base of the ciliary shaft. The transition zone appears to apply different gating mechanisms to safeguard the functional specificity of the ciliary compartment. Consistently, many ciliopathy-associated gene mutations affect transition zone proteins (Gonçalves and Pelletier, 2017). First, the transition zone appears to constitute a membrane diffusion barrier, with a ciliary zone of exclusion that prevents non-ciliary membrane proteins from entering the PC, but also maintains ciliary membrane proteins within the PC compartment (Williams et al., 2011; Cevik et al., 2013; Jensen and Leroux, 2017). Additionally, the transition fibres and transition zone appear to establish a soluble diffusion gate, in the way of a molecular sieve. Indeed, studies using a permeabilised system for ciliary trafficking in mammalian cells have reported that proteins of increasing size fused to GFP do not enter the PC with the same dynamics: while proteins below 4.8 nm enter the PC, entry is decreased for proteins between 4.8 and 8.6 nm, and is no longer detectable for larger proteins (Breslow et al., 2013). Similarly, diffusion of fluorescent proteins established a ciliary sieve-like barrier allowing the entry of soluble proteins with a Stokes radius as large as 7.9 nm (Lin et al., 2013). The precise molecular mechanisms involved in this sieve remain however elusive. A similarity with the nuclear pore complex (NPC) has been proposed, with studies revealing the implication of the nuclear transport machinery in ciliary trafficking (Dishinger et al., 2010; Fan et al., 2011; Kee et al., 2012), although some diffusion events may occur independently (Breslow et al., 2013).

This membrane and soluble diffusion barrier at the base of the PC allows the separation between the cytoplasm and the cilioplasm, and is essential for the functional specialisation of the ciliary antenna as an extracellular signal sensor. Consistently, studies have challenged the view that small second messenger signals (*e.g.*, cAMP and cGMP cyclic nucleotides, calcium), locally produced within the PC compartment in response to the activation of ciliary membrane receptors, can freely diffuse between the cytoplasm and cilioplasm (Delling et al., 2016; Jiang et al., 2019), and argue in favour of a ciliary compartmentalisation of second messenger signals, that signal and function independently from the cytoplasmic pool. Indeed, in FRET experiments, Moore and colleagues reported that in inner medullary collecting duct cells (IMCD3), primary cilia have a high basal cAMP concentration with regards to the cytoplasm (∼5 times higher; Moore et al., 2016). In another study, pharmacological inhibition of the ciliary-localised vasopressin receptor type-2 in kidney epithelial cells induced increased cilioplasmic, but not cytoplasmic, cAMP levels. Conversely, fluid-shear stress decreased cilioplasmic cAMP levels, without affecting the cytoplasmic pool (Sherpa et al., 2019). In the case of cGMP, studies in *Caenorhabditis elegans* olfactory sensory neurons expressing a genetically encoded cGMP indicator show that, following odour exposure, ciliary cGMP levels transiently decreased, while cGMP levels in dendrites and soma gradually increased (Shidara et al., 2017). Similar observations have also been reported for calcium (Nauli et al., 2008; Delling et al., 2013; Jin et al., 2014; Sanchez et al., 2023; Shim et al., 2023). At the functional level, ciliary *versus* extra-ciliary second messenger signals have been reported to regulate different signalling pathways and mechanisms. For example, optogenetic increase of ciliary cAMP levels in zebrafish developing somites was shown to inhibit Hedgehog signalling, while cytoplasmic cAMP levels did not (Truong et al., 2021). Similarly, in developing zebrafish embryos, ciliary PKA, by contrast to cytosolic PKA, was found to specifically regulate the Hedgehog pathway (Zhang et al., 2024). In line with these observations, Hansen and colleagues unravelled a ciliary cAMP signalosome that is functionally distinct from the cytoplasm and drives kidney cyst formation (Hansen et al., 2022). Moreover, during cortical interneuron migration, ciliary cAMP and cGMP signals were found to antagonise each other to regulate cell polarity, while centrosome-located cAMP and cGMP acted in synergy to control another aspect of migration, which is nucleokinesis (Atkins et al., 2023b). Similar reports have been made concerning calcium, unravelling the PC as a calcium-mediated mechanosensory compartment that is necessary and sufficient to instruct left-right asymmetry during zebrafish development (Djenoune et al., 2023).

### 2.2 The primary cilium: a key signalling platform for neuronal guidance signalling pathways

Among the variety of signalling pathways and cell functions regulated by the PC signalling hub, receptors for some of the major signalling pathways that are involved in neuronal guidance processes have been found.

The first major evidence establishing the PC as a key signalling compartment in neuronal development arose in 2003 from a forward genetic screen conducted by Huangfu and colleagues in mouse embryos. They discovered that genes encoding intraflagellar transport machinery proteins are essential for embryonic ventral patterning through the signalling of Sonic hedgehog (Shh; Huangfu et al., 2003), one of the most important morphogens involved in neuronal development (Douceau et al., 2023). Since this pioneer study, the ciliary transduction of the Shh pathway–most commonly referred to as the canonical pathway (Teperino et al., 2014) – has been described (Rohatgi et al., 2007), and its role in neuronal development extensively reviewed (Bangs and Anderson, 2017). Since then, several components of the Shh transduction machinery have been localised to neuronal primary cilia (Figure 1, right-hand), such as the Patched receptor for Shh and the Smoothened (Smo) GPCR (a key signal transducer of the Shh pathway) in the PC of rat hippocampal neurons, or GPR161, which is a negative regulator of Shh canonical signalling (Mukhopadhyay et al., 2013), in the PC of dI1 commissural neurons (Petralia et al., 2011; Toro-Tapia and Das, 2020). Notably, Shh signalling at the PC has been involved in several neuronal guidance processes, including neuronal migration (Baudoin et al., 2012; Pedraza et al., 2024) and axon pathfinding (Dumoulin et al., 2024).

But the role of the PC in neuronal guidance processes is not limited to the transduction of the Shh signalling pathway. Another major guidance molecule, Wnt, primarily identified as a guidance molecule for navigating commissural axons in the mammalian spinal cord (Lyuksyutova et al., 2003) and subsequently involved in neuronal migration (Boitard et al., 2015; Bocchi et al., 2017), has been linked to the PC. The Wnt signalling pathway comprises a network of various signalling molecules, with Wnt ligands often activating frizzled receptors together with an array of different co-receptors. Two main branches of the pathway are classically distinguished: the canonical Wnt/β-catenin pathway and the non-canonical Wnt/PCP pathway. Signalling molecules of the Wnt transduction machinery have been found to localise to the PC of non-neuronal cells (*e.g.*, Dishevelled, β-catenin, LRP5/6). Among these, some have been reported in the primary cilia of neurons. Such is the case, for example, of Frizzled-1, expressed in the PC of developing olfactory sensory neurons (Rodriguez Gil and Greer, 2008). The transmembrane Frizzled-like receptor Tmem67/MKS-3, a transition zone protein that functionally binds Wnt5a (Abdelhamed et al., 2015) and whose mutations are responsible for the MKS and JBTS ciliopathies, has moreover been located to the PC base of the *C*. *elegans* ciliated sensory neurons (Williams et al., 2010). It has further been shown to regulate canonical Wnt/β-catenin signalling in the developing cerebellum (Abdelhamed et al., 2019). However, the relationship between PC and Wnt signalling is complex. While Wnt signalling can regulate ciliogenesis, the PC can regulate Wnt signalling. Moreover, the question of whether the PC structure is required for the activation and transduction of the Wnt/β-catenin signalling pathways is controversial (Anvarian et al., 2019; Vuong and Mlodzik, 2023; Niehrs et al., 2025). Of note, several ciliary signalling components of the Wnt pathway are not exclusively localised to the PC. Such is the case of Frizzled-1, which has also been found in dendrites and axons of developing olfactory sensory neurons (Rodriguez Gil and Greer, 2008), highlighting the need for further studies to distinguish ciliary from extra ciliary regulations of Wnt-associated processes.

In addition to the Shh and Wnt pathways, extensively studied for their ciliary transduction, key molecular players in neuronal guidance pathways classically studied for their role in growth cones, have also been linked to the PC compartment. Immunohistochemistry experiments performed in migrating cortical interneurons have indeed identified several guidance receptors at the ciliary surface, namely, the TrkB receptor for BDNF (Brain-derived neurotrophic factor), the GFRα-1 receptor for GDNF (glial cell line-derived neurotrophic factor), CXCR4 and CXCR7 receptors for the CXCL12 chemokine, the ErbB4 receptor for Neuregulin1 (NRG-1), serotonin receptor 6 (5HT6), receptors Robo1 and 2 for Slit, and the MET receptor for HGF/SF (hepatocyte growth factor/scatter factor; Higginbotham et al., 2012). In addition to these receptors, an *in vivo* BioID (iBioID) proteomic screen has recently revealed in the PC of adult neurons (Loukil et al., 2023) the presence of Ephrin (involved both in neuronal migration and axon guidance processes) and GABA-A and GABA-B receptors, involved in synaptogenesis (Fiorentino et al., 2009; Sui et al., 2024) and neuronal migration (Heck et al., 2007). Finally, the receptor tyrosine kinase PDGFR-α (Clement et al., 2013), the CD44 hyaluronan receptor (Jones et al., 2012; Lee et al., 2020) and neuropilin 1 (Pinskey et al., 2017), all involved in different neuronal guidance processes (see sections below), have also been localised to the PC of non-neuronal cells. Future studies will be crucial to unravel how this multitude of ciliary signalling receptors regulate specific steps of neuronal guidance, in a cell type and cell stage specific manner.

Together, these studies pinpoint the neuronal PC as a key subcellular signalling compartment in neuronal guidance, integrating a variety of extracellular cues at the crossroads between different guidance processes. The downstream signalling effectors activated by ciliary guidance receptors, and how they regulate guidance processes, remain however obscure. This is mostly due to the technological challenge that represents the dissection of the ciliary-specific functions of guidance signalling receptors/effectors, with dual subcellular localisation (*i.e.*, ciliary and extra-ciliary). Yet, during the past decade, some labs have developed innovative strategies to tackle this issue and provided important new insights into the molecular mechanisms underlying the PC-elicited regulation of neuronal guidance pathways. In the following sections, we will review our current knowledge of PC function in neuronal migration and axon guidance, and discuss future avenues to be explored.

## 3 Primary cilium signalling in neuronal migration

### 3.1 The primary cilium compartment in neuronal migration

A role for the PC in the acquisition of cell polarity and directed cell migration has long been established in various non-neuronal systems (Christensen et al., 2013; Veland et al., 2014). In fibroblasts, for example, the PC–together with the centrosome–re-orients prior to the initiation of migration (Katsumoto et al., 1994) and is then oriented parallel to the direction of the movement (Albrecht-Buehler, 1977). Furthermore, the PC genetic ablation abrogates chemical or electrical stimuli-evoked directed cell migration in fibroblasts or mesenchymal stem cells (Schneider et al., 2005; 2010; Pruski et al., 2016; 2019; Lee et al., 2020; Nakazato et al., 2023). Mutation of a ciliopathy-associated gene was also found to induce neural crest cell migration defects in the zebrafish model (Tobin et al., 2008). Despite such evidence, a role for primary cilia in neuronal migration has remained vaguer and more controversial, with some data reporting PC formation in the neocortex only after neuroblast migration has occurred, and no PC involvement in the establishment of neuronal polarity, neuronal migration or cortical laminar organisation (Arellano et al., 2012). By contrast, other groups have reported a role for primary cilia in the apico-basal polarity of radial glial cells (Higginbotham et al., 2013), in the tangential migration of cortical interneurons (Baudoin et al., 2012; Higginbotham et al., 2012), as well as in neuroblasts migrating postnatally through the rostral migratory stream towards the olfactory bulb (Matsumoto et al., 2019; Stoufflet et al., 2020). Strengthening the decisive role of the PC in neuronal migration, several gene mutations responsible for neurodevelopmental disorders–including ciliopathies or focal malformations of cortical development–and affecting ciliogenesis have been reported to impair radial or tangential neuronal migration in the developing cortex (Guo et al., 2015; Park et al., 2018).

### 3.2 Guidance cue-evoked primary cilium molecular pathways in neuronal migration

Neuronal migration is a well-documented cyclic saltatory process (Bellion et al., 2005; Schaar and McConnell, 2005; Tsai and Gleeson, 2005). In the first step of the cycle, migrating neurons probe their surroundings by extending and stabilising a leading process in an attractive or permissive environment. The centrosome then moves forwards to a proximal region within this process, called the dilatation or swelling compartment, before the nucleus dynamically translocates towards the centrosome in a process termed nucleokinesis. In 2012, Baudoin and colleagues showed that the PC genetic ablation altered the ability of interneurons migrating *ex vivo* in brain organotypic slices to exit their tangential migration stream and invade their target destination (*i.e.*, the developing cortical plate), in a way that mimics Shh pathway inhibition, suggesting a role for Shh-initiated PC signalling in neuronal migration (Baudoin et al., 2012). The same year, Higginbotham and colleagues identified by immunohistochemistry experiments many guidance cue receptors in the PC of migrating cells (*i.e.*, TrkB, GFRα-1, CXCR4, CXCR7, ErbB4, 5HT6, Robo1 and 2, MET). Using a microfluidic device, they moreover cultured cortical interneurons and dorsal cortical cells in two opposite chambers linked by microlanes, allowing to expose the cortical interneurons of one chamber to a gradient of migration-regulating cues secreted by the dorsal cortical cells of the other chamber. Using this setup, the authors further revealed that PC-ablated cortical interneurons (*i.e.*, interneurons carrying a null-mutation for the small regulatory GTPase Arl13b) exhibit defective migration towards the source of the gradient, compared to wild-type interneurons (Higginbotham et al., 2012). These two pioneer studies have opened the exciting and complex question of how the activation of guidance receptors at the PC may regulate the different steps of neuronal migration: what are the specific downstream signalling events and cellular processes regulated by these PC-dependant guidance signals?

**FIGURE 2:**
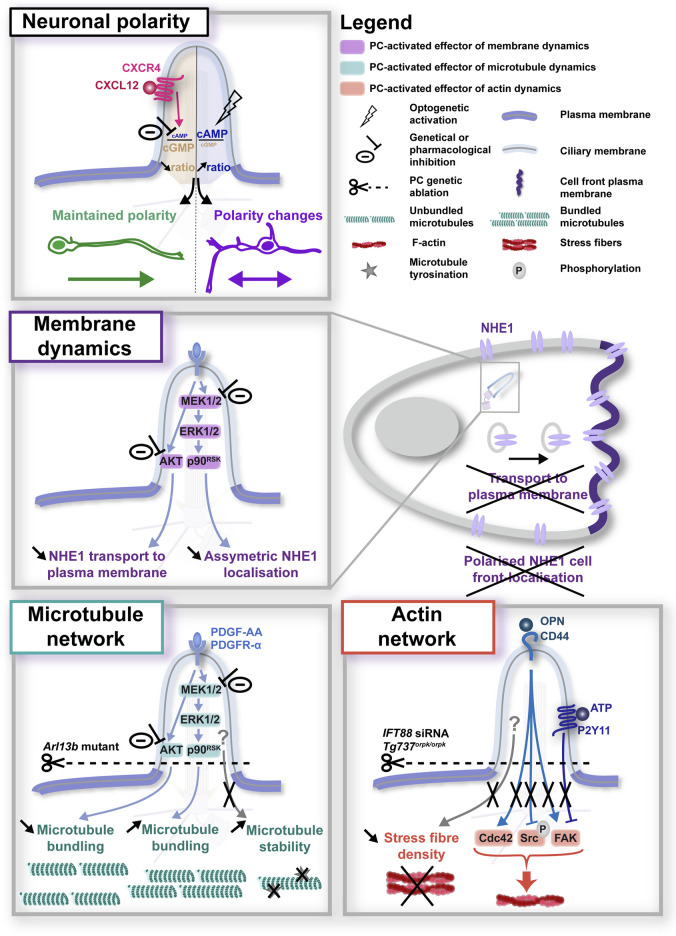
Primary cilium-elicited signalling pathways in neuronal migration. Top: in neurons, regulation of the ciliary cAMP/cGMP ratio downstream of CXCL12/CXCR4 activation at the PC surface was found to regulate the cell polarity and direction of migrating cells (top), although the downstream effectors activated in the cytoplasm remain to be identified (Atkins et al., 2023b). Middle and bottom summarise the research on downstream cytoplasmic effectors performed in migrating non-neuronal cells, that converge on the regulation of membrane dynamics (middle; Clement et al., 2013) or the microtubule (left-hand bottom; Clement et al., 2013; Pruski et al., 2016) and actin network (right-hand bottom; Jones et al., 2012; Mansini et al., 2019; Lee et al., 2020). In all panels, experimental manipulations (genetic, optogenetic or pharmacological) performed to alter ciliary signals, together with their phenotypic consequences, are colour-coded in black.

Very few studies have started to tackle this question. In a study performed in tangentially-migrating mouse neurons in the postnatal rostral migratory stream, genetic ablation of the PC led to altered nucleokinesis of migrating neurons, in a mechanism dependent on a centrosome-located cAMP hotspot, thereby linking the PC regulation of migration to a downstream centrosomal component (Stoufflet et al., 2020). Recently, the same group proposed a ciliary pathway involving GPR161 mechanosensitivity as the upstream trigger regulating the centrosomal cAMP hotspot and the organisation of the nuclear cage of microtubules, required for proper nucleokinesis to occur (Paillard et al., 2025). Given the wide range of guidance receptors expressed at the ciliary surface, linking specific PC-elicited guidance signals to specific downstream effectors and migratory behaviours remains however challenging. The fact that many ciliary membrane receptors are also expressed at the extra-ciliary plasma membrane further complexifies the situation, highlighting the need to develop new tools to bypass loss of function approaches and alter PC-elicited signals specifically at the ciliary compartment. Using newly developed genetically encoded molecular tools targeted to the PC to selectively modulate (*i.e.*, increase or buffer) PC-elicited second messenger signals, combined with live cell imaging and pharmacological/genetic approaches, Atkins and colleagues recently added some pieces to the puzzle. They showed that the CXCL12 chemokine controls the cell polarity and branching behaviour of migrating cortical interneurons by decreasing the ciliary cAMP/cGMP ratio upon binding to its CXCR4 receptor (Atkins et al., 2023b; Figure 2, top). Such technological development paves the way towards the dissection of the specific role on migratory behaviours of other guidance receptors present at the ciliary surface, and to the identification of their specific downstream molecular effectors.

Precious clues may be gained from studies already linking ciliary molecular mechanisms to cell migration in non-neuronal cells. Interestingly, in such systems, PC-elicited signals have been reported to impact cell migration through the regulation of various mechanisms.

#### 3.2.1 The primary cilium and the regulation of membrane dynamics

One of those mechanisms concerns **the regulation of membrane dynamics** (Figure 2, middle). In a study conducted by the Christensen lab in fibroblast cells, the Platelet-Derived Growth Factor AA (PDGF-AA) protein activated the PI3K-AKT and MEK1/2-ERK1/2-p90^RSK^ pathways at the PC, and inhibiting these pathways counteracted the ability of PDGF-AA to stimulate migration in scratch-assay experiments (Clement et al., 2013), corroborating previous studies from the group (Schneider et al., 2005; 2010). Moreover, Clement et al. found that PDGF-AA signalling at the PC activates the Na^+^/H^+^ exchanger NHE1 and is critical for directed migration. More precisely, they show that while AKT inhibition impedes NHE1 vesicles from reaching the plasma membrane, inhibition of MEK1/2 abolishes the preferential localisation of NHE1 to the plasma membrane of the cell front, with cells displaying a broader NHE1 membrane distribution in multiple membrane locations (Clement et al., 2013). This study builds upon a previous study from the group involving NHE1 in directed cell migration downstream of ciliary PDGF-AA signalling (Schneider et al., 2009), and is in agreement with other studies establishing a role for NHE1 in cell migration and invasion (Cardone et al., 2005; Stock and Schwab, 2006; Stock and Pedersen, 2017), through various mechanisms, such as the regulation of cell polarity by anchoring actin filaments to the cell front plasma membrane (Denker and Barber, 2002). Of note, this role for ciliary MEK1/2 activation in NHE1 asymmetric membrane localisation is highly coherent with the well-established role of the PC in cell polarity and directed migration, also reported in migrating neurons (Atkins et al., 2023b). Together, these data open the possibility of a role for the PC in the regulation of the cell front behaviour through the control of membrane dynamics and/or the targeting of specific receptors to the plasma membrane (Figure 2). Interestingly, the PDGFR-α receptor for PDGF-AA has been found expressed in migrating neurons of the external germinal layer (EGL) of the cerebellum (Andrae et al., 2001). However, although it has been involved in the migration of astrocytes (Itoh et al., 2011), its role in neuronal migration remains uncharacterised. On the other hand, NHE1 has been involved in the migration and invasive behaviour of cancer cells in glioblastoma (Cong et al., 2014), as well as in early neurite outgrowth during neuronal development (Sin et al., 2009; 2020). To our knowledge, its regulation of neuronal migration has so far not been described, let alone downstream of neuronal PC activation. Thus, while they appear as attractive candidate players in PC-dependant cell migration, future studies will be required to determine whether PC-elicited guidance pathways, PDGFR-α-NHE1-related or -independent, may regulate membrane dynamics to control cell polarity or plasma membrane composition in a context of neuronal migration.

#### 3.2.2 The primary cilium and the regulation of cytoskeletal dynamics

Another key cell process reported in non-neuronal migrating cells downstream of PC-elicited pathways is the **regulation of cytoskeletal dynamics** (Figure 2, bottom). Very few studies have analysed the effect of PC signalling on microtubule dynamics. The Christensen lab has nevertheless reported defects in extra-ciliary microtubule bundling downstream of PDGF-AA signalling at the PC (Clement et al., 2013), in addition to an effect of an *Arl13b* null mutation on microtubule detyrosination (*i.e.*, a post-translationnal modification that correlates with a more stable state of microtubules) reported by Pruski and colleagues in mouse embryonic fibroblast cells (Pruski et al., 2016). By contrast, more studies have addressed the question of a role for the PC on actin dynamics during cell migration, with the identification of different F-actin regulators activated by PC signalling during cell migration. First, genetic ablation of the PC by siRNA-mediated knockdown of the intraflgellar transport 88 (IFT88) protein was found to abolish the phosphorylation of **focal adhesion kinase** (FAK, a tyrosine kinase that functions as a signalling scaffold for the assembly and maturation of the focal contacts regulating cell adhesion), that occurs in response to osteopontin (OPN) signalling at the PC in wild type migrating mesenchymal stem cells (Lee et al., 2020). A similar decrease in FAK phosphorylation following PC genetic ablation (deletion of intraflagellar transport protein Tg737: *Tg737* ^*orpk/orpk*^) was observed in endothelial cells, in association with a decreased directionality of migrating cells (Jones et al., 2012). Moreover, in migrating cholangiocytes, ATP stimulation of the ciliary purinergic receptor P2Y11 induced a rapid degradation of FAK in ciliated cells, which was abolished in de-ciliated cells (Mansini et al., 2019). Another F-actin regulator targeted by PC signalling is the **Src kinase**, whose phosphorylation dynamics are disrupted in migrating cells upon PC genetic ablation compared to controls, whether in basal conditions or following OPN signalling (Lee et al., 2020). Of note, the same study reported an increased expression of **the Cdc42 Rho GTPase** in IFT88-silenced cells. Finally, and in addition to these different actin regulators, the PC has been suggested to regulate **the stress fibre network** of migrating endothelial cells, which regulates several functions in migrating cells, such as the generation of traction forces, the maturation of integrin-based adhesions, the establishment of cell polarity (Vicente-Manzanares et al., 2009). Intriguingly, studies report a reduction of the actin stress fibres observed in mutated endothelial cells displaying impaired PC assembly (*Tg737* ^*orpk/orpk*^), compared to controls (Jones et al., 2012). To our knowledge, the P2Y11 purinergic receptor and the CD44 surface hyaluronan receptor (for OPN) have not been localised to neuronal primary cilia. However, independently of the PC, CD44 has been involved in the migration of neural precursor cells (Deboux et al., 2013). Similarly, purinergic receptors have been involved in neuronal migration or axon guidance (Rodrigues et al., 2019), although the P2Y11 receptor has not been reported so far in such processes.

Importantly, microtubule and F-actin remodelling are well established as key driving forces of neuronal migration (Schaar and McConnell, 2005; Shan et al., 2021) and axon guidance (Sánchez-Huertas and Herrera, 2021; Atkins et al., 2023a). Consistently, several guidance receptors found by the Anton lab in the PC of migrating cortical interneurons (Higginbotham et al., 2012; see Figure 1) are known to regulate membrane or cytoskeletal dynamics in a PC-independent context. These data highlight the need to dissect whether and how guidance signals elicited in neuronal primary cilia regulate cytoskeletal remodelling and/or membrane/receptor trafficking to drive specific migratory or axon steering behaviours.

## 4 Primary cilium signalling in axon guidance

### 4.1 The primary cilium compartment in axon guidance

Evidence of a role for the PC in axon navigation processes came from axonal tract defects observed in patients. Indeed, several ciliopathies (*i.e.*, Joubert, Meckel Gruber, Acrocallosal and Orofacial Digital Syndromes) have been associated with a defective development of the corpus callosum (CC; Salonen, 1984; Odent et al., 1998; Holub et al., 2005; Takanashi et al., 2009; Poretti et al., 2011; Putoux et al., 2011), which consists in the largest axonal tract of the brain, formed by millions of axons that connect homologous cortical areas of the two brain cerebral hemispheres. Consistently, in Joubert Syndrome, defects of other major axonal tracts, displaying failure to cross the midline, have also been reported, such as the corticospinal tract (CST; Poretti et al., 2007; Théoret et al., 2013) and the superior cerebellar peduncle (SCP) tract (Spampinato et al., 2008). Of note, the molar tooth sign, characterised by thickened and elongated SCPs that fail to cross the midline, is one of the hallmarks of Joubert Syndrome and related disorders (Maria et al., 1999; Sattar and Gleeson, 2011; Romani et al., 2013). Defective decussation, fasciculation and/or branching of axonal tracts–including the SCP, CST, CC tracts and developing sensory corneal nerves–has also been reported in mouse models of Joubert syndrome and related disorders (Guo et al., 2019) or following the conditional knockout of the ciliopathy-associated *IFT88* gene (Portal et al., 2019). Additionally, abnormal projection of thalamocortical axons towards the amygdala was reported in two ciliary mouse mutants (Magnani et al., 2015). Similarly, RNAi silencing of the Joubert Syndrome gene *C5orf42* in chick embryos led to pathfinding defects of the commissural dI1 axons (Asadollahi et al., 2018). Corroborating these studies, in a genetic screen based on the *in utero* electroporation of a library of 30 shRNA targeting ciliopathy-linked genes in the cortex of E14,5 mouse embryos, Guo and colleagues identified aberrant axonal trajectory and fasciculation of neurons depleted for BBS5, BBS7, BBS9, BBS11, BBS12 and TMEM216 (Guo et al., 2015). Of note, changes in the adhesion properties of a developing neuron are likely to modify the way its axon will interact with other axons and/or cells from the surrounding environment, in a complex manner that can lead to axon guidance defects. Consistently, in the case of BBS5 and BBS7 knockdown, the authors moreover report defective axonal midline crossing towards the contralateral cortex, with miss-directed axons that, instead of crossing, project aberrantly towards subcortical targets once they have reached the midline.

### 4.2 Guidance cue-evoked primary cilium molecular pathways in axon guidance

**FIGURE 3:**
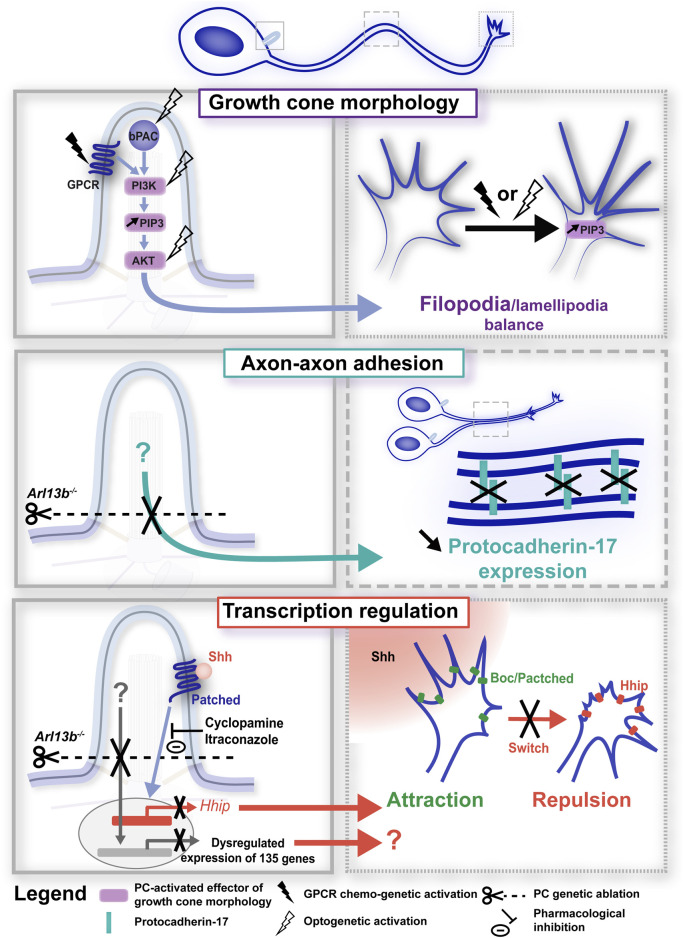
Primary cilium-elicited signalling pathways in axon pathfinding. Signalling pathways elicited at the PC (left-hand boxed regions, full line) induce phenotypic changes at the axonal and/or growth cone compartments (right-hand boxed region, large and small dotted lines, respectively). PC-elicited signalling pathways have been found to regulate axon pathfinding dynamics through the regulation of growth cone morphology (top; Guo et al., 2019), axon-axon adhesion (middle; Guo et al., 2019) and transcription (bottom; Guo et al., 2019; Dumoulin et al., 2024). In all panels, experimental manipulations (genetic, chemo-genetic, optogenetic or pharmacological) performed to alter ciliary signals, together with their phenotypic consequences, are colour-coded in black. Left- and right-hand boxed regions correspond to a higher magnification of the PC (full line), axon (large dotted line) or growth cone (small dotted line) compartments of the developing neuron depicted above. GPCR, G-protein coupled receptor; bPAC, bacterial (*Beggiatoa*) photoactivated adenylyl cyclase; Shh, Sonic hedgehog.

While some of these axonal tract defects have been shown to occur in a non-cell autonomous manner, as a result of the defective distribution of glial and neuronal guide post cells (Benadiba et al., 2012; Laclef et al., 2015; Putoux et al., 2019), studies have also identified a cell autonomous role for the ciliary compartment in the regulation of axon pathfinding, involving different PC-elicited signalling pathways. In a study performed by the Anton lab, the conditional knockdown of the Joubert Syndrome-associated gene *Arl13b* in cultured deep cerebellar nuclei (DCN) neurons led to reduced dynamic axonal branching, aberrant growth cone morphology with altered filopodia-lamellipodia balance (*i.e.*, numerous longer filopodial protrusions), as well as impaired axon-axon adhesion associated with reduced recruitment of the protocadherin-17 (Pcdh17) to axon-axon contacts (Guo et al., 2019; Figure 3, bottom and middle). Interestingly, these axonal and growth cone morphological defects were associated to an increase in the ciliary levels of the PIP3 second messenger. Using elegant tools based on the CIBN/CRY2 dimerization optogenetic system, Guo and colleagues showed that recruiting PIP3 or AKT to the PC of DCN neurons is sufficient to alter growth cone morphology and dynamics by inducing filopodial protrusions. They further use DREAAD chemo-genetic tools to show that modulating the activity of ciliary G-protein coupled receptors GPCRs (that are known to converge onto PIP3) recapitulates the PIP3-AKT-linked growth cone morphological defects (Figure 3, top). Together, these data highlight PIP3-AKT as a PC-elicited signalling pathway involved in growth cone remodelling and behaviour.

Given that the PC, that is organised by the centrosome, is located near the cell soma and consequently at a distance from the axonal growth cone, such results raise the question of the ciliary downstream molecular effectors and mechanisms that propagate the signals down the axon to the exploring growth cone. Interestingly, Guo and colleagues observed a gradual increase in PIP3 activity at the growth cone of DCN neurons following ciliary PIP3 activation (Guo et al., 2019; Figure 3, top), and propose that positive feedback networks involving kinase-dependent cascades may rapidly spread locally-induced PC signalling over long distances. Following RNA-seq analyses in E12.5 *Arl13b* ^*−⁄−*^ and control embryos, they further propose PC-induced regulation of transcriptional programs as an additional mechanism to regulate axon navigation processes (Figure 3, bottom). Their identification in ciliary mutants of differentially expressed genes involved (among other processes) in cell adhesion opens the possibility that the defective Pcdh17-mediated axon-axon adhesion observed in Arl13b conditional knockout neurons may be due to altered gene transcription. In agreement, the Stoeckli lab has recently identified a role for the PC of developing chick commissural axons in mediating a transcriptional switch of Shh receptors, required to elicit the well-documented behavioural switch (from attraction to repulsion) of commissural axons crossing the midline (Dumoulin et al., 2024). In chick dI1 neurons, the authors indeed showed that *IFT88* silencing impaired dI1 axon midline crossing in a cell autonomous manner. *IFT88* silencing was moreover associated in *in situ* hybridisation experiments with a reduced expression of the Hhip (hedgehog-interacting protein) receptor, which is required for the repulsive response to Shh and the rostral turn of post-crossing commissural axons (Bourikas et al., 2005; Wilson and Stoeckli, 2013). Importantly, preventing Smo entry in the PC in response to Shh activation, pharmacologically or genetically (using a hSmoCLD construct that prevents Smo ciliary localisation after endogenous Smo silencing), led to misprojecting commissural dI1 axons or reduced Hhip expression, respectively, supporting the requirement of Shh signalling at the PC for the induction of *Hhip* transcription and correct dI1 axon guidance (Dumoulin et al., 2024; Figure 3, bottom). This elegant study further opens the question of whether additional mechanisms required for axon guidance may be regulated by the PC, such as the axonal transport or exocytosis of Hhip at the growth cone membrane. In addition to a role for canonical Shh signalling in mediating gene transcription required for axon guidance, a non-canonical Shh pathway (*i.e.*, that is transcription independent) that relies on the PC has been reported in the axonogenesis of chick postmitotic neurons (Toro-Tapia and Das, 2020). In developing chick embryos, neuroepithelial cells undergoing proliferation have been reported to delaminate from the neuroepithelium as they exit the cell cycle. Postmitotic neurons then initiate axon outgrowth and navigation for the formation of functional neuronal circuits. In this study, authors showed that as neuroepithelial cells delaminate, the PC is disassembled through apical abscission, followed by a PC re-assembly at the onset of axonogenesis. Preventing ciliary re-assembly by chromophore-assisted light inactivation impaired the axonogenesis of newborn neurons by inducing axonal collapse. Using a Gli reporter construct, authors further observed that canonical Shh signalling (*i.e.*, Gli activity-dependent) in the PC is lost upon delamination, and is no longer observed in the newly assembled PC. Although this newly-assembled PC gradually displayed Smo accumulation (suggestive of Shh signalling), immunostaining revealed the presence of the GPR161 negative regulator of canonical Shh signalling. Finally, pharmacological inhibition of the Src family kinases, which mediate the cytoskeletal rearrangements downstream of non-canonical Shh signalling, induced axon collapse, supporting a model in which the re-assembled PC is required for axonogenesis by mediating non-canonical Shh signalling. Whether the non-canonical Shh signalling is also required during growth cone turning events, in addition to axon extension, remains to be uncovered.

Taken together, these studies show to what extent guidance signalling pathways initiated in the ciliary compartment close to the soma influence the axon and growth cone behaviours required for accurate axon navigation. It is interesting to note that a long-distance influence of ciliary signals was also reported to regulate the branching behaviour of the leading process in the case of neuronal migration (Atkins et al., 2023b). Further studies will be required to precisely unravel the molecular effectors linking ciliary signals to axonal and growth cone behavioural remodelling.

## 5 Conclusion: Insights to be gained from ciliary guidance pathways for our understanding of the aetiology of neurodevelopmental disorders

The increasing interest for the once-neglected ciliary compartment initially arose from the discovery of its involvement in a wide range of disorders. Indeed, in addition to ciliopathies, a dysfunction of the PC has now been involved in different neurodevelopmental (*e.g.*, schizophrenia, autism spectrum disorder, bipolar disorder, intellectual disability …) and neurodegenerative disorders (Valente et al., 2014; Kaliszewski et al., 2015; Youn and Han, 2018; Park et al., 2019; Hasenpusch-Theil and Theil, 2021; Karalis et al., 2022; Ma et al., 2022; Volos et al., 2025), as well as in cancer cell migration/invasion (Eguether and Hahne, 2000; Higgins et al., 2019), including glioblastoma (Álvarez-Satta and Matheu, 2018). Conversely, studying the PC-elicited signalling pathways and molecular mechanisms regulating guidance processes in physiological conditions now appears as a key step to better understand the aetiology of such disorders. Interestingly, our increasing knowledge of PC-elicited guidance signalling and its functional and molecular versatility, both refines and complexifies our understanding of the role of this tiny organelle in pathology, at multiple levels.

First, the PC can regulate multiple aspects of a same neuronal guidance process. For example, during cell migration, the PC controls membrane dynamics, cytoskeletal dynamics but also focal adhesion dynamics. This occurs either through the activation of different ciliary membrane receptors (*e.g.*, PDGFR-α, P2Y11, CXCR4), or through the activation of a same ciliary receptor (*e.g.*, PDGFR-α) that can regulate multiple cellular mechanisms (*e.g.*, membrane and microtubule dynamics, see Figure 2), sequentially or concomitantly through the activation of several parallel downstream pathways.

Second, a same ciliary signalling molecule can be involved in different stages of neuronal guidance. For example, in the genetic screen performed by Guo and colleagues, silencing of the Bardet-Biedl Syndrome-associated *BBS7* gene led to a disrupted apical-basal polarity of radial glial cells, but also to a defective multipolar to bipolar transition of migrating principal neurons, and to altered axonal trajectory and fasciculation of cortical neurons (Guo et al., 2015). Likewise, while Shh appears to regulate the migration of developing cortical interneurons (Baudoin et al., 2012), it is also involved in the extension and navigation of developing axons, either through the transcriptional regulation of key guidance receptors (Dumoulin et al., 2024), or through a non-canonical pathway involving Src kinase activation (Toro-Tapia and Das, 2020).

Third, the presence of multiple guidance receptors both at the PC and growth cone surface highlights the importance of understanding the specific function of guidance receptor activation at each subcellular compartment. For example, guidance receptors such as Robo1/2 and ErBb4 have been linked to neurodevelopmental disorders, such as Autism Spectrum Disorder for Robo1/2 (Anitha et al., 2008) and schizophrenia and bipolar disorder for ErbB4 (Iwakura and Nawa, 2013; Mei and Nave, 2014). Identifying the specific contribution of the compartmentalised ciliary signalling of these receptors appears crucial in this context to better apprehend the complexity of such disorders and gain new insigths into their aetiology and treatment. Likewise, in cancer cell migration, while CXCL12 (Guo et al., 2016; Luo et al., 2019; Hayasaka et al., 2022) and Ephrin (Campbell et al., 2006; Wang, 2011; Cho et al., 2018) signalling have been linked to metastasis, the specific role on invasive behaviour of their local signalling at the ciliary compartment remains poorly characterised. Unravelling PC-elicited signalling pathways and downstream molecular effectors may therefore provide precious clues for future translational studies aiming to identify new therapeutic targets specific to PC signalling in order to selectively correct specific cell behaviours (*i.e.,* invasion).

Understanding the specific role of the identified ciliary guidance receptors (see Figure 1) in different steps of neuronal guidance is a crucial step of this complex process. The complexity of the task lies in the diversity of ciliary receptors, that are not always exclusive to the ciliary compartment. Rising to this challenge will critically rely on the use and development of new tools to selectively manipulate (*i.e.*, block/activate) specific membrane receptors located exclusively at the ciliary surface (without affecting the other ciliary receptors through PC genetic ablation, for example,) or their downstream second messenger signals. Such genetic, chemo-genetic and optogenetic tools are already starting to emerge to selectively buffer endogenous ciliary second messenger signals or trigger specific second messenger signalling within the ciliary compartment (Guo et al., 2019; Hansen et al., 2022; Atkins et al., 2023b).
